# Efficacy of topotecan in pretreated metastatic poorly differentiated extrapulmonary neuroendocrine carcinoma

**DOI:** 10.1002/cam4.807

**Published:** 2016-07-25

**Authors:** Leonidas Apostolidis, Frank Bergmann, Dirk Jäger, Eva Caroline Winkler

**Affiliations:** ^1^ Department of Medical OncologyNational Center for Tumor DiseasesUniversity Hospital HeidelbergHeidelbergGermany; ^2^ Institute of PathologyUniversity Hospital HeidelbergHeidelbergGermany

**Keywords:** chemotherapy, NEC, neuroendocrine carcinoma, topotecan, unknown primary

## Abstract

Therapeutic options for metastatic poorly differentiated neuroendocrine carcinoma (NEC) after prior platinum‐based chemotherapy are limited. Topotecan is an approved second‐line chemotherapy for small cell lung cancer (SCLC). NEC is often considered to show a biological behavior similar to SCLC. The aim of this study was to analyze the efficacy of topotecan in pretreated metastatic NEC patients. We performed a retrospective analysis of all patients treated with topotecan for metastatic NEC who presented at our center between January 2005 and December 2014 (*n* = 30). All 30 patients had received at least a platinum and etoposide containing regimen as prior chemotherapy. Median proliferation rate (Ki67) was 80%. As best response to topotecan five patients showed a stable disease, two patients a partial remission, resulting in a disease control rate of 23%. Of the remaining 23 patients, 14 (47%) showed a progressive disease, nine (30%) died before radiologic response could be evaluated. Median progression‐free (PFS) and overall survival (OS) after start of topotecan was 2.1 and 4.1 months, respectively. In the subgroup analysis, patients with unknown primary (vs. those with a known primary) showed a significantly prolonged PFS of 3.5 months (vs. 1.9, *P* = 0.0107) and OS of 6.7 months (vs. 2.6 months, *P* = 0.0168). Grade 3/4 hematotoxicity was observed in 60% of patients. Topotecan shows only moderate antitumor activity in metastatic NEC. Disease control rate is lower than reported for SCLC. However, antitumor activity of topotecan seems higher in patients with unknown primary.

## Introduction

Poorly differentiated extrapulmonary neuroendocrine carcinomas (NECs) are an aggressive subset of neuroendocrine neoplasms. Primary tumors can arise in different organs, including the gastrointestinal tract, the genitourinary tract and the head and neck region; in 7–30% of patients the site of the primary remains unknown 1, 2, 3. According to the proliferation rate‐based grading in the current WHO classification, NECs with a gastrointestinal primary are defined by a proliferation rate >20% 4. There is a current debate based on preliminary data indicating that NECs are a heterogeneous disease, and those with a lower proliferation rate (e.g., 20–60%) might need to be treated differently from those with a higher proliferation rate 5, 6, 7. Poorly differentiated NECs are considered to show a similar biologic behavior to small cell lung cancer (SCLC). For example, both metastatic NEC and SCLC are commonly treated with platinum and etoposide‐based regiments 5, 8, 9, 10. Prognosis for NEC patients remains poor with a median progression‐free survival of 9 months and overall survival of 6–19 months. Therapeutic options after progression on first‐line therapy are limited and have only been analyzed retrospectively so far 5, 7, 11, 12, 13, 14.

Topotecan, a topoisomerase I inhibitor, is an approved second‐line chemotherapy for SCLC 15, 16, 17, 18. It is often used in NEC treatment because of the similarities between SCLC and NEC and also recommended in treatment guidelines despite the lack of published data for this concept so far 19.

The aim of this study was to analyze the efficacy of topotecan in pretreated metastatic NEC patients.

## Patients and Methods

We analyzed retrospectively all patients who were treated with topotecan for metastatic NEC and presented at the Department of Medical Oncology at the National Center for Tumor Diseases, University Hospital Heidelberg, Germany between January 2005 and December 2014. Patients were identified through electronic patient's record. All histopathological findings were reviewed by the investigators to comply with the diagnostic criteria of the most current 2010 WHO classification 4. As standard of care, topotecan was administered intravenously at a dose of 1.25 mg/m² per day on days 1–5, the cycle was repeated every 22 days. Tumor assessment was performed every 8 to 12 weeks.

The duration of the therapy as well as the response were recorded, progression‐free survival (PFS) and overall survival (OS) were calculated. PFS was defined as the time span between the start of the topotecan therapy and the date of progression or death due to any cause, OS was defined as the time length between start of topotecan treatment and the date of death from any cause.

Significant toxicities, defined as grade 3–4 according to the National Cancer Institute Common Toxicity Criteria (NCI‐CTC), were recorded as well as necessary dose reductions.

After the end of topotecan therapy patients were followed during regular follow‐up visits and further therapies.

Statistical analysis was carried out with GraphPad Prism, Version 6.05 (GraphPad Software, La Jolla, USA). Median survival was estimated using the Kaplan–Meier method, differences in survival were analyzed using the log‐rank test. A p‐value below 0.05 was considered significant.

The trial was approved by the institutional research ethics committee (approval S‐428/2014).

## Results

### Patient characteristics

A total of 30 patients with NECs treated with topotecan could be identified (Table 1). The median follow‐up was 42.7 months, one patient was lost to follow‐up after documented disease progression. All patients had received at least a platinum and etoposide containing regimen (PE) as prior chemotherapy. Three patients had received both cisplatin and carboplatin. Five patients (17%) had received an additional therapy regimen. The median proliferation rate (Ki67) was 80%. Nine tumors (30%) were reported positive for thyroid transcription factor 1 (TTF‐1), 15 (50%) were negative. Primary tumors included gastrointestinal (esophagus, stomach, colorectal, pancreas) (47%), urogenital (ureter, bladder, prostate) (13%), and unknown origin (40%). Of the 12 patients with unknown primary, pulmonary metastases were detected in three cases. In one case the primary in the esophagus was classified as mixed adenoneuroendocrine carcinoma (MANEC); however, histologic workup of the metachronous metastases showed only neuroendocrine differentiation.

**Table 1:** Patient characteristics

	Number of patients (*n* = 30)	%
Age [years]		
Median	59	
Range	41–76	
Sex		
Male	23	77
Female	7	23
ECOG performance status		
0	11	37
1	15	50
2	3	10
3	1	3
Primary tumor		
Unknown	12	40
Pancreas	3	10
Stomach	3	10
Esophagus	2	7
Colorectal	6	20
Prostate	2	7
Bladder/ureter	2	7
Ki67 [%]		
Median	80	
Range	30–100	
20–30%	2	7
31–60%	3	10
61–100%	21	70
Not reported	4	13
TTF‐1		
Positive	9	30
Negative	15	50
Not reported	6	20
Histology		
Large cell	16	53
Small cell	14	47
Metastatic sites		
Median number	3	
Range	1–5	
Lymph nodes	23	77
Liver	21	70
Bone	12	40
Lung	10	33
Brain	5	17
Peritoneum	3	10
Other	5	17
Prior therapy		
Median lines	1	
Range	1–4	
Cisplatin + etoposide	19	63
Carboplatin + etoposide	15	50
Other^a^	5	17
Best response to PE		
CR	0	0
PR	10	33
SD	6	20
PD	13	43
Duration of response to PE		
<90 days	25	83
≥90 days	5	17

CR: complete remission, ECOG: Eastern Cooperative Oncology Group, PD: progressive disease, PE: platinum + etoposide, PR: partial remission, SD: stable disease, TTF‐1: thyroid transcription factor‐1.

a Other prior therapies included oxaliplatin + 5‐fluorouracil (FOLFOX), FOLFOX + cetuximab, epirubicin + oxaliplatin + 5‐fluorouracil (EOF), FOLFOX followed by docetaxel followed by temozolomide, and sunitinib followed by everolimus.

### Efficacy

**Figure 1:**
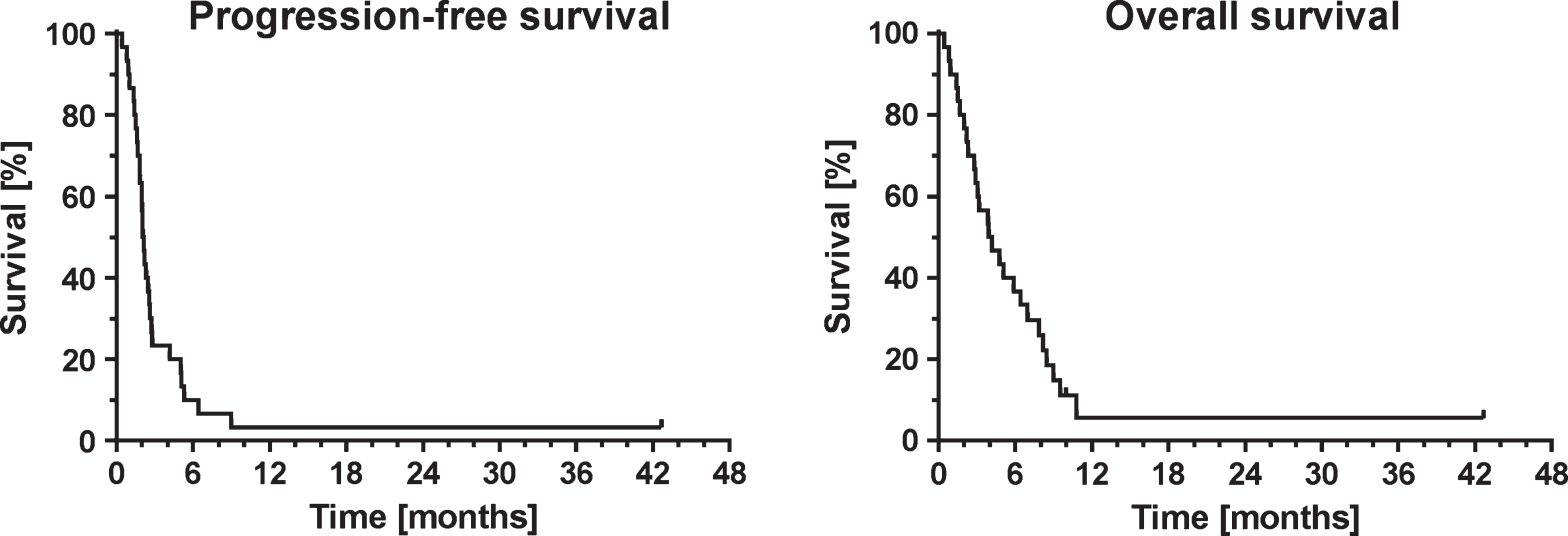
Kaplan–Meier plots of PFS and OS of all patients (*n* = 30) treated with topotecan.

A median of two cycles of topotecan were applied (range 1–8) (Table 2). There were no documented cases of complete remission (CR). As best response, partial remission (PR) could be achieved in two patients (7%), stable disease (SD) in five patients (17%), resulting in a disease control rate of 23%. Fourteen patients (47%) showed radiologically documented progressive disease (PD), nine patients (30%) died before response could be radiologically evaluated due to clinical progression. Median progression‐free survival (PFS) and overall survival (OS) was 2.1 and 4.1 months, respectively (Fig. 1). Eight patients (27%) received subsequent therapy after progression to topotecan, including doxorubicin + cyclophosphamide + vincristine (ACO) in three cases, sunitinib in one case, paclitaxel in one case, carboplatin + paclitaxel in one case, re‐exposition with carboplatin + etoposide in one case and finally FOLFOX followed by carboplatin + gemcitabine in one case.

**Table 2:** Results of topotecan treatment

	Number of patients (*n* = 30)	%
Cycles of topotecan		
Median	2	
Range	1–8	
Best response to topotecan		
PR	2	7
SD	5	17
PD	14	47
Death before response evaluation	9	30
Median Survival [months]		
PFS	2.1	
OS	4.1	

PD: progressive disease, PFS: progression‐free survival, PR: partial remission, OS: overall survival, SD: stable disease.

**Figure 2:**
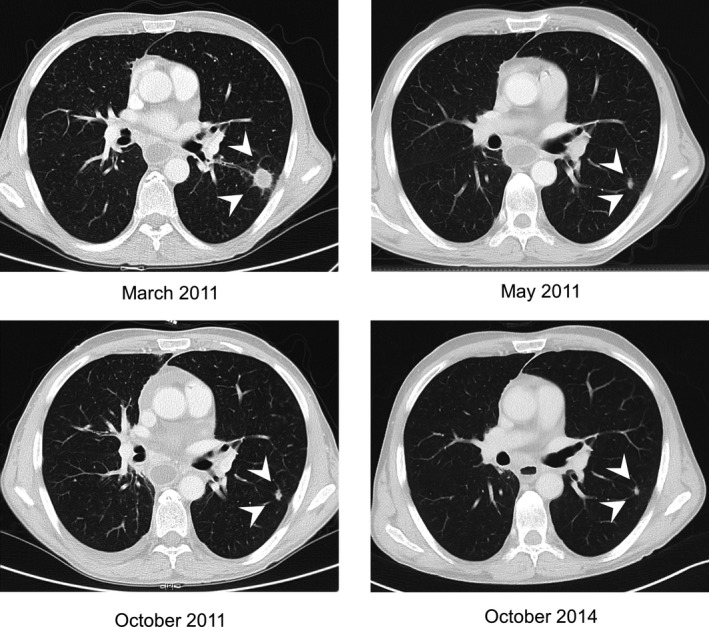
Computed tomography scans of a 49‐year‐old patient treated with topotecan after progression to platinum and etoposide. The patient was diagnosed with cervical and mediastinal lymph node metastases of neuroendocrine carcinoma in September 2010. At this timepoint, no suspicious pulmonary lesions could be detected. After 5 cycles of chemotherapy with cisplatin and etoposide as well as irradiation of the cervical and mediastinal lymph nodes the patient showed a new pulmonary metastasis in March 2011 (white arrowheads). From April until October 2011 the patient received a total of 8 cycles of topotecan resulting in a sustained PR without signs of tumor activity for more than 3 years.

Both cases with documented PR had an unknown primary. Most notably, one of those showed a prolonged PR under eight cycles of topotecan and is still in follow‐up for over 3 years without signs of new disease activity (Fig. 2).

**Figure 3:**
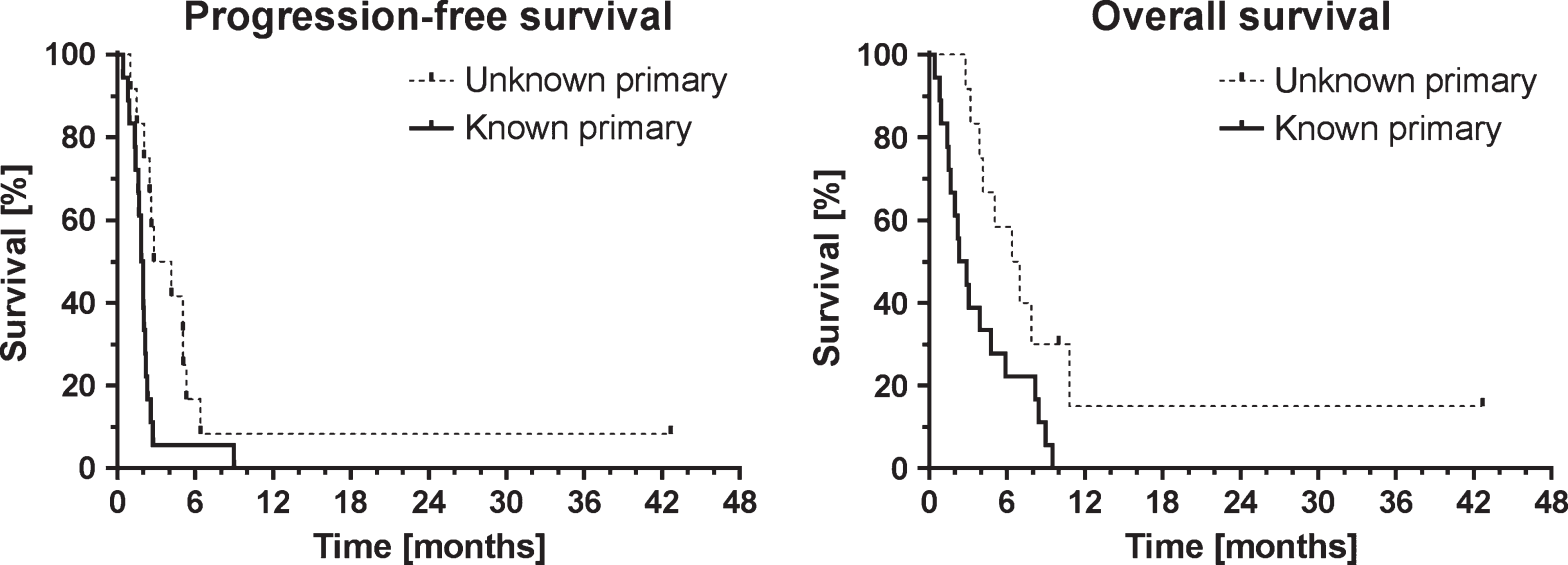
Kaplan–Meier plots of PFS and OS for the subgroups of patients with unknown (*n* = 12) and known (*n* = 18) primary.

When looking at the different subgroups, those patients with an unknown primary (vs. those with a known primary) showed a significantly prolonged PFS of 3.5 months (vs. 1.9 months, *P *=* *0.0107) and OS of 6.7 months (vs. 2.6 months, *P *=* *0.0168) (Fig. 3).

Of the other analyzed subgroups, there was no significant difference in PFS or OS with respect to performance status, large cell or small cell histology, proliferation rate (Ki67), TTF‐1 immunohistochemistry, duration of disease control under PE, or response to PE (Table 3). However, better performance status and TTF‐1 positivity showed a trend for a prolonged OS.

**Table 3:** Median OS and PFS for different patient subgroups

	PFS [months]	*P*	OS [months]	*P*
Primary				
Known	1.9	0.0107	2.6	0.0168
Unknown	3.5		6.7	
ECOG Performance Status				
0	2.5	0.9656	6.4	0.1530
1	1.8		3.9	
≥2	2.4		2.9	
Histology				
Small cell	2.0	0.6452	3.4	0.2115
Large cell	2.2		5.6	
Ki67 [%]				
<60	2.2	0.4679	2.2	0.4078
≥60	2.0		4.1	
Best response to PE				
PR	1.8	0.1873	2.8	0.3300
SD	2.6		5.9	
PD	2.1		3.9	
Duration of response to PE [days]				
<90	2.1	0.7584	3.9	0.9038
≥90	4.2		5.1	
TTF‐1				
Positive	2.3	0.4807	4.2	0.1159
Negative	2.1		3.1	

ECOG: Eastern Cooperative Oncology Group, PE: platinum + etoposide, PFS: progression‐free survival, OS: overall survival.

### Toxicities

The main toxicity attributed to topotecan treatment was decreased bone marrow function. Grade 3/4 hematotoxicity was observed in 18 patients (60%), including anemia in 12 (40%), thrombocytopenia in 9 (30%), and neutropenia in 14 patients (47%). Grade 3/4 infections (including febrile neutropenia) occurred in nine patients (30%). Dose reduction had to be performed in 10 patients (33%).

## Discussion

To our knowledge, our study is the largest analysis of a single chemotherapy regimen in pretreated NEC patients so far. Intravenous topotecan showed only modest antitumor activity in pretreated metastatic NEC considering the fact that topotecan as second‐line treatment after failure of platinum‐based regimens is a common practice in the oncological treatment of NECs. Significant hematotoxicity was observed in the majority patients. A recently published study of 22 patients treated with oral topotecan showed very similar results regarding PFS and OS, however, no objective responses were noted and the subgroup analysis could not identify a group of patients who could benefit more from topotecan 20. Hematotoxicity was lower than in our study. Both oral and intravenous applications of topotecan have also been deemed equally effective in SCLC 15, 17.

For topotecan in SCLC, response rates und disease control rates have been reported with 7–26% and 36–51%, respectively, median PFS and OS with 2.7–3.4 months and 5.8–8.1 months, respectively 15, 16, 18. These values are considerably higher than in our study with NEC patients. Despite the postulated biological similarities between SCLC and NEC, several reports have noticed significant differences in pathological and moleculargenetic features, pathogenesis, and response to therapy 21, 22, 23, 24, 25. However, results comparing the clinical course are inconsistent: some studies show a better prognosis for SCLC 21, some for NEC 23, 25. The main reason for this could be the heterogeneity of the NEC cohort. Numerous studies have shown that clinical course among NECs vary greatly (including survival as well as response to first‐line and second‐line therapy) according to performance status, location of the primary, and proliferation rate 5, 6, 7.

In our subgroup analysis, antitumor activity seemed to be increased for NECs with unknown primary. Most notably, both patients with documented PR had an unknown primary, one with an unusual long response. Pulmonary metastases could only be detected in three of the 12 patients with unknown primary and were clearly not evident as primary tumors. However, autopsy studies for cancer of unknown primary revealed the lung as one of the most common primary sites 26. One could speculate that a small pulmonary primary with a possible higher sensitivity to topotecan might go undetected and therefore account for the better outcome. Positivity for TTF‐1, a marker considered a characteristic of pulmonary neuroendocrine neoplasms, led only to a trend in prolongation of OS without reaching statistical significance. However, TTF‐1 positivity in poorly differentiated extrapulmonary NECs varies greatly from 7 up to 84%, therefore questioning the value of TTF‐1 to discriminate between pulmonary and extrapulmonary primary in NECs 27, 28, 29, 30, 31, 32, 33, 34, 35, 36.

Other retrospective studies with temozolomide‐based regimens 7 as well as with 5‐fluorouracil and irinotecan (FOLFIRI) 11 showed higher disease control rate (71% and 57%, respectively) and median survival (OS 22 and 18 months, respectively) than topotecan. An important difference, however, is that the NECs in our analysis had a higher median proliferation rate than in these previous studies. In the temozolomide study, only nine (36%) of the patients had a Ki67 >60%. Response rates for these patients were described as lower, although not reported in detail. In the FOLFIRI study only five patients (26%) had a Ki67 >60%. Of these patients, as best responses to FOLFIRI, 1 showed PD, 1 PR and the remaining 3 SD. Median PFS and OS of these patients was 6.0 and 16.0 months, respectively.

Recent reports on the anthracyclin amrubicin showed some antitumor activity in platinum refractory NECs with a PFS of up to 3.7 and an OS of up to 7.8 months 12, 13, 14, 37. The overall response rate was up to 39%.

In the Nordic NEC study, 100 patients are reported to have received a second‐line chemotherapy, mainly with temozolomide‐based (35 patients) and docetaxel‐based regimens (20 patients) 5. OS (counting from start of first‐line therapy) was 19.0 months. However, further details like OS counting from start of second‐line therapy or response rates as well as characteristics of these 100 patients were not reported.

In summary, the optimal therapeutic strategy for NEC is still to be defined. Although all discussed therapeutic regimens (topotecan, temozolomide‐based, FOLFIRI, amrubicin, docetaxel‐based) show some antitumor activity in pretreated metastatic NEC, the data result from retrospective analysis so far. Furthermore, due to the heterogeneous tumors summarized under the category of NEC, comparison between these regimens is difficult. Especially in higher proliferative NECs activity of all mentioned regimens is limited.

A first step to improve treatment outcome for NEC patients could be to characterize subgroups systematically, for example, according to the proliferation rate. Our study shows that the location of the primary tumor might be an important factor influencing chemotherapy response. Based on these subgroups, prospective randomized trials including the therapeutics mentioned above as well as novel treatment modalities are needed.

## Conflicts of interest

None declared.
